# Circular RNA circDUS2 Is a Potential Biomarker for Intracranial Aneurysm

**DOI:** 10.3389/fnagi.2021.632448

**Published:** 2021-05-19

**Authors:** Xin Chen, Shuzhe Yang, Junhua Yang, Qingyuan Liu, Maogui Li, Jun Wu, Hao Wang, Shuo Wang

**Affiliations:** ^1^Department of Neurosurgery, Beijing Tiantan Hospital, Capital Medical University, Beijing, China; ^2^China National Clinical Research Center for Neurological Diseases, Beijing, China; ^3^Center of Stroke, Beijing Institute for Brain Disorders, Beijing, China; ^4^Beijing Key Laboratory of Translational Medicine for Cerebrovascular Diseases, Beijing, China

**Keywords:** circular RNA, intracranial aneurysm, circDUS2, circRNA microarray, smad

## Abstract

**Background:** CircRNAs have been found to play a crucial role in the pathological process of various kinds of diseases. However, the role of circRNAs in the formation and rupture of intracranial aneurysm is still unknown.

**Methods:** Differentially expressed circRNAs profiles between superficial temporal arteries (*n* = 5) and intracranial aneurysms (*n* = 5) were analyzed using the Arraystar human circRNAs microarray. Quantitative real-time PCR was utilized to validate the differential expression of circDUS2. Fluorescence *in situ* hybridization (FISH) was meant for the location of circDUS2 in human brain vascular smooth muscle cell (HBVSMC). Structural analysis was used to speculate on the function of circDUS2.

**Results:** Five hundred forty-three upregulated and 397 downregulated significantly in intracranial aneurysm as compared to superficial temporal arteries. Quantitative real-time PCR verified the elevated expression of the upregulated circDUS2. The FISH test revealed that circDUS2 is located in the cytoplasm of brain vascular smooth muscle cells.

**Conclusion:** This study showed differential expression data of circRNAs between superficial temporal artery and intracranial aneurysm and revealed that circDUS2 is a potential molecular marker for intracranial aneurysm.

## Introduction

Intracranial aneurysm (IA) is abnormal bulging brought about by structural damage to intracranial artery walls because of multiple factors. Depending on the latest epidemiological research, the morbidity of IA is 8% (Vlak et al., 2011). The rupture of IA can pose a serious threat to human life and health. The mortality of IA rupture within 30 days is nearly 50% and 30% of survivors suffered moderate to severe disabilities (Lawton and Vates, 2017). The assessment of the possibility of rupture is essential to the therapy of IA. Existing research speculated that inflammation, family heredity, geometrical morphology of IA, and hemodynamics are related to the reconstruction of the aneurysm wall, but the details are far from clear. In recent years, the molecular mechanism of aneurysm occurrence and rupture has been studied deeply; inflammation, apoptosis, phenotypic changes of vascular smooth muscle cells, cell adhesion, atherosclerosis, and abnormal extracellular matrix metabolism may be involved in the rupture mechanism of IA (Jiang et al., 2018; Jabbarli et al., 2020). Therefore, the exploration of the mechanism of IA rupture and the search for reliable molecular targets are of great significance for the prediction and diagnosis of IA rupture in advance, guiding the selection of treatment strategies for unruptured aneurysms, directing primary and secondary prevention, and reducing mortality and disability rates. Circular RNA (CircRNA) is one of a specific type of non-coding RNA. It used to think to be a low abundance RNA due to the incorrect splicing of the exon transcript. In recent years, as the use of the RNA sequencing technique became widespread, it has been found that many exon transcripts can accept non-linear reverse splicing or gene rearrangement to form circRNA (Chen and Yang, 2015). Research on the biological function of circRNA and the effect of human diseases has just started (Chen et al., 2017). Some characteristics of circRNA have been disclosed. It is broadly expressed in human cells (Salzman et al., 2012) and is hard to degrade by exonuclease (Memczak et al., 2013); most of them are located in the cytoplasm instead of the cell nucleus (Zhang et al., 2013). Functions of circRNAs also have been explored, such as microRNA sponges (Hansen et al., 2013), which interact with different proteins (Du et al., 2017) and even encode functional peptides or proteins (Pamudurti et al., 2017; Yang et al., 2017). It is becoming increasingly clear that circRNAs play a crucial role in the pathological process of many kinds of cancers, such as colorectal cancer (Zeng et al., 2018), breast cancer (Pamudurti et al., 2017), and hepatocellular carcinoma (Yang et al., 2017). However, research about the role of circRNAs in the formation and rupture of intracranial aneurysm is rare, and the overall pathophysiological contributions of circRNAs to intracranial aneurysm remain largely unknown. In the present study, we used the circRNA microarray to acquire circRNA profiles in human IA tissues as compared to superficial temporal artery tissues. Subsequently, we performed bioinformatical analysis to explore the potential functions of circRNA in IA. Thus, these data would lay a foundation for future investigations on the molecular functions of circRNAs in IA.

## Materials and Methods

### Patients and Specimens

This study was approved by the Medical Ethics Committee of Beijing Tiantan Hospital, Capital Medical University. Every patient admitted in this research has written informed consent. IA specimens were obtained from 15 IA patients undergoing aneurysm clipping. Superficial temporal artery specimens were collected from 15 matched patients without IAs. Among them, five pairs of STA and IA samples were used to conduct a circRNA microarray analysis. Other samples were used to perform qRT-PCR. All these specimens were suspended in liquid nitrogen immediately.

### Total RNA Isolation and Quality Control

Total RNA was extracted from five paired STA tissues and IAs with the use of a Trizol Reagent (Invitrogen). The NanoDrop ND-1000 (Thermo Fisher Scientific, Wilmington, DE, USA) was utilized to test the purity and concentration of RNA (Supplementary Table 1). We excluded the contamination of DNA. The integrity of the total RNA was achieved by electrophoresis on a denatured agarose gel.

### RNA Labeling and Hybridization

Sample labeling and array hybridization were performed according to the manufacturer's protocol (Arraystar Inc.). Briefly, total RNAs were digested with Rnase R (Epicenter, Inc.) to remove linear RNAs and rich circular RNAs. Then, the enriched circular RNAs were amplified and transcribed into fluorescent cRNA utilizing a random priming method (Arraystar Super RNA Labeling Kit; Arraystar). The labeled cRNAs were purified by an RNeasy Mini Kit (Qiagen). The concentration and specific activity of the labeled cRNAs (pmol Cy3/μg cRNA) was measured by NanoDrop ND-1000. One microgram of each labeled cRNA was fragmented by adding a 5 μl 10 × blocking agent and 1 μl of a 25 × fragmentation buffer, then the mixture was heated at 60°C for 30 min, and finally a 25 μl 2 × hybridization buffer was added to dilute the labeled cRNA. Fifty microliters of hybridization solution was dispensed into the gasket slide and assembled to the circRNA expression microarray slide. The slides were incubated for 17 h at 65°C in an Agilent Hybridization Oven. Hybridized arrays were washed, fixed, and scanned using the Agilent Scanner G2505C.

### circRNA Microarray Analysis

Agilent Feature Extraction software (version 11.0.1.1) was used to analyze the acquired array images. Quantile normalization and subsequent data processing were performed using the R software limma package. Differentially expressed circRNAs with statistical significance between two groups were identified through Volcano Plot filtering. Differentially expressed circRNAs between two samples were determined through Fold Change filtering. Hierarchical Clustering was performed to show the distinguishable circRNAs expression pattern among samples.

### Quantitative Real-Time PCR

Five upregulated circRNAs were selected for further investigation. Quantitative real-time PCR was utilized to verify these differential expressions. We have achieved total RNA of IAs and STA tissues. RNase R (Lucigen, 20U, 37°C, 3 h) was used to purify the circRNAs again. The relevant cDNAs were composed (M-MLV, Promega) and stored in −20°C. QuantStudio5 Real-Time PCR System (Applied Biosystems) was used to perform qRT-PCR. The sequence of circRNA results was obtained from the database “circBase” (http://circrna.org). Primers were obtained by RiboBio (Guangzhou, China) (Supplementary Table 3). Owing to the influence of concentration quantitative error and reverse transcription efficiency error, the cDNA content of every sample was different. In order to correct these errors, we regarded housekeeping gene β-actin as an internal reference; as a result, we accepted the ratio of genes to be tested and the internal reference, in other words, the relative content of the gene to be tested.

### GO and KEGG Pathway Analysis

The GO enrichment analysis divides gene functions into three aspects: cellular components (CCs), molecular functions (MFs), and biological processes (BPs). After we find out our target circRNAs, miRNAs binding on our target circRNAs also can be found. We perform GO analysis on parental genes of these miRNAs with R. The *P*-value after adjustment represents the significance of GO terms. We also perform a KEGG pathway analysis of parental genes of circRNA-binding miRNAs in order to reveal the biological or pathological processes in which circRNAs participate. The *P*-value after adjustment represents the significance of pathway correlations as well.

### Statistical Analysis

The fold changes were estimated by unpaired Student's *t*-test and used to identify the differentially expressed circRNAs in the sample of IAs. CircRNA was selected as differentially expressed with a *P* < 0.05 and a fold change >1.5, which means they were statistically significant. The significance of qRT-PCR was evaluated by Student's *t*-test, and *P* < 0.05 was considered statistically significant; it was analyzed by GraphPad Prism 8.4.0 (GraphPad Software, La Jolla, CA, USA). Other statistical methods such as chi-squared test, Wilcoxon signed-rank test, and Mann–Whitney *U*-test were also performed. All statistical analyses were done by SPSS 19.0 (SPSS, Inc., Chicago, IL).

## Results

### Identification of circRNA Microarray in Human IA and STA Samples

**Figure 1:**
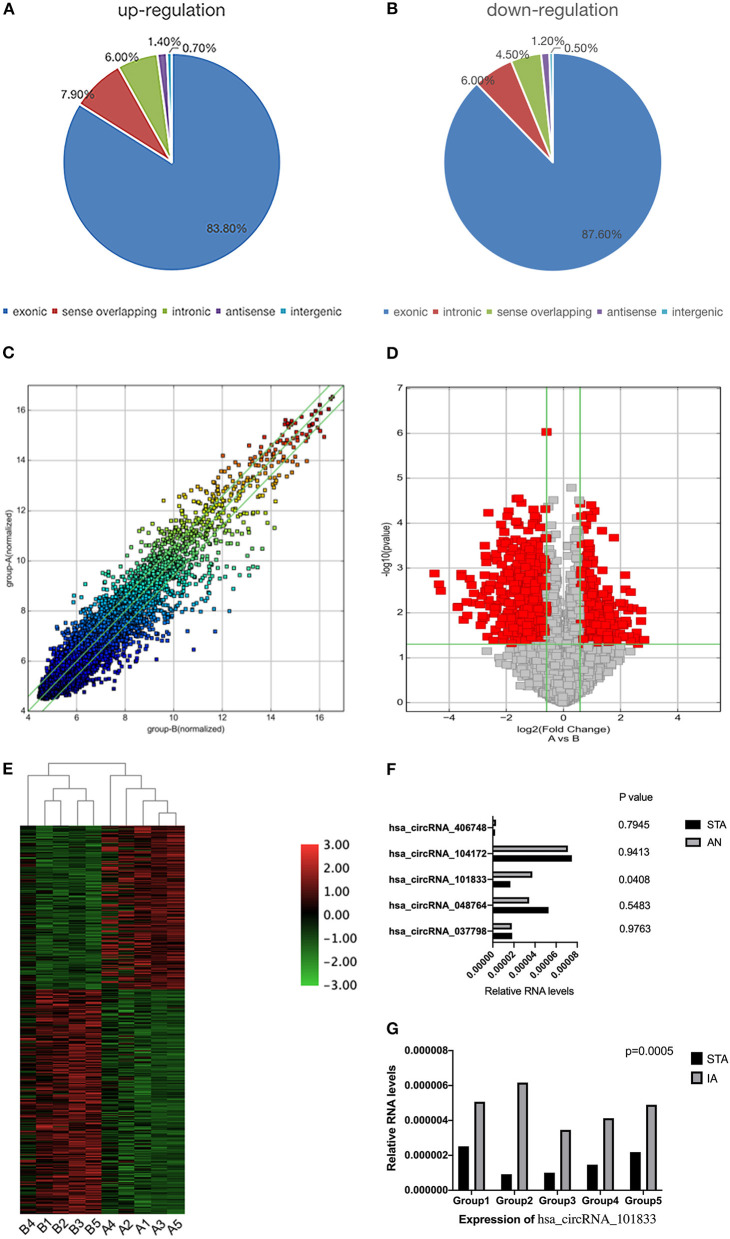
Differential expression of circRNAs in IA tissues. **(A)** The constituent of upregulated circRNAs. **(B)** The constituent of downregulated circRNAs. **(C)** The scatterplot is used for assessing the circRNA expression variation between the two compared samples or two compared groups of samples. The values of X and Y axes in the scatterplot are the normalized signal values of the samples (log_2_ scaled) or the averaged normalized signal values of groups of samples (log_2_ scaled). The green lines are Fold Change Lines. The circRNAs above the top green line and below the bottom green line indicate more than 1.5-fold change of circRNAs between the two compared samples. **(D)** Volcano Plots are used for visualizing differential expression between two different conditions. The vertical lines correspond to 1.5-fold up and down, respectively, and the horizontal line represents a *p*-value of 0.05. So the red point in the plot represents the differentially expressed circRNAs with statistical significance. Group A represented STA samples; group B represented IA samples. **(E)** The hierarchical clustering of differentially expressed circRNAs. “Red” indicates high relative expression, and “green” indicates low relative expression. Group A represented STA samples; group B represented IA samples. **(F)** Validation of the differential expression of five upregulated circRNAs. **(G)** Validation of the differential expression of hsa_circRNA_101833 in another five coupled groups. STA, superficial temporal arteries; IA, IA.

We detected a total of 13,174 circRNAs using the Arraystar human circRNA Microarray (Supplementary Table 4). Among them, 942 circRNAs dysregulated between STA and IA tissues (fold change > 1.5; *P* < 0.05), and 750 of them have been identified in circBase by other studies. Furthermore, comparing IA with superficial temporal artery, 544 circRNAs upregulated while 398 of them downregulated (Supplementary Table 5). All circRNAs were classified into five types: “exonic,” “intronic,” “antisense,” “sense overlapping,” and “intergenic.” Among the upregulated circRNAs, 456 (83.82%) circRNAs consist of exons, 43 (7.9%) circRNAs transcribed from the same gene locus as the linear transcript but not classified into “exonic” and “intronic” were classified as sense overlapping, 33 (6.07%) were intronic, 8 (1.47%) were antisense, and 4 (0.74%) were intergenic (Figure 1A). For downregulated circRNAs, there were 349 (87.69%) exonic, 24 (6.03%) intronic, 18 (4.52%) sense overlapping, 5 (1.26%) antisense, and 2 (0.5%) intergenic (Figure 1B). CircRNA expression variations between the two compared groups of samples were assessed (Figure 1C). The Volcano Plot was constructed through fold change values and *p*-values and used for visualizing the differential expression between the STA and IA samples (Figure 1D). Hierarchical clustering revealed the circRNA expression in IAs and the superficial temporal artery (Figure 1E). For further investigation, we selected five circRNAs upregulated in aneurysm samples (hsa_circRNA_104172, hsa_circRNA_048764, hsa_circRNA_037798, hsa_circRNA_406748, and hsa_circRNA_101833). Besides fold change > 1.5 and *P* < 0.05, their raw intensities in both the IA and STA groups were more than 200, and their parental genes were well-investigated by other researchers in order to reveal these circRNAs' functions better. We perform qRT-PCR in another five paired STA and IA samples to verify the circRNA microarray profiling expression results. The results showed that two of the five circRNAs that we have selected upregulated in IA, but only the overexpression of hsa_circRNA_101833 was significant (*P* < 0.05; Figure 1F). Then, five more paired samples were used to verify the differential expression of hsa_circRNA_101833 in two groups, and the result was identical to the microarray analysis and the qRT-PCR performed for the first time (Figure 1G).

### Characteristics and Functions of circDUS2

**Figure 2:**
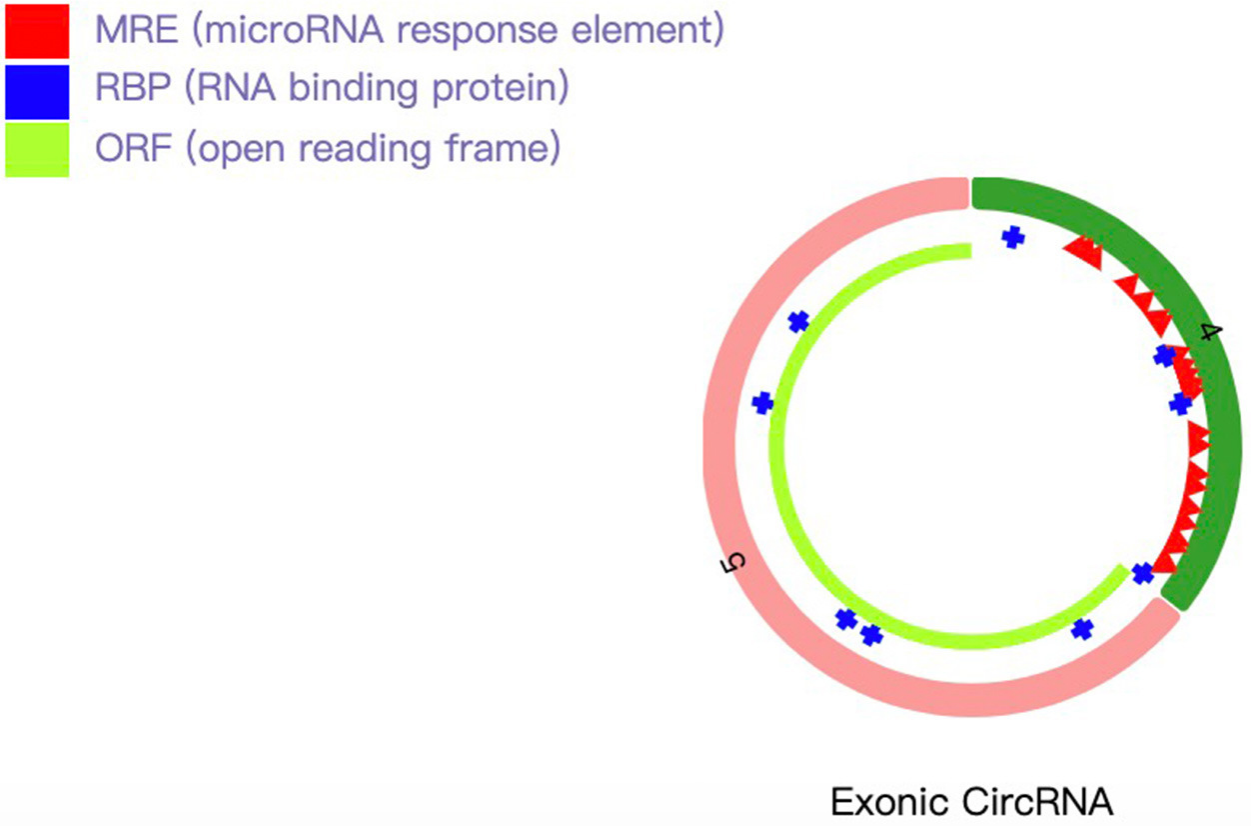
Schematic diagram of hsa_circRNA_101833. The light green bar represents the open reading frame, the blue bar represents proteins binding with hsa_circRNA_101833, and the red bar represents the microRNA binding sites.

hsa_circRNA_101833 is derived from exon 4 and exon 5 of the DUS2 gene, and its CircBase ID is hsa_circ_0039908. hsa_circRNA_101833 contains an open reading frame (ORF). MicroRNA binding sites also have the ability of binding protein (Figure 2). To determine whether this ORF is functional, we have achieved the nucleotide sequence of hsa_circRNA_101833 from CircBase, and then three ORFs were detected by ORFfinder (https://www.ncbi.nlm.nih.gov/orffinde) (Table 1). Among the three ORFs, only the longest one has the capacity to encode protein. The results of the Conserved Domains (https://www.ncbi.nlm.nih.gov/Structure/cdd/wrpsb.cgi) showed that. The ORF may encode the triose phosphate isomerase (TIM) superfamily (Bit Score = 56.73, *E*-value = 4.13e-12). According to previous researches, TIM is a dimeric, non-allosteric enzyme of the glycolytic pathway and catalyzes the interconversion of the three-carbon sugars dihydroxyacetone phosphate (DHAP) and D-glyceraldehyde 3-phosphate (GAP) (Wierenga et al., 2010). Another function of circRNA is as microRNA sponges, which means to control gene transcription by binding with microRNAs. To elucidate the function of microRNA sponges, miRNAs connected with hsa_circRNA_101833 were found (CSCD, http://gb.whu.edu.cn/CSCD/) and listed in Table 2. Furthermore, we recognized proteins binding with hsa_circRNA_101833 from CSCD, and the result is listed in Table 3.

**Table 1:** Open reading frames detected from hsa_circRNA_101833.

**Label**	**Strand**	**Frame**	**Start**	**Stop**	**Length (nt|aa)**	**Nucleotide sequence**
ORF1	+	3	99	>224	126|41	MILNSLSLCYHNKLILAPMVRVGTLPMRLLALDYGADIVYCE
ORF2	–	1	131	45	87|28	MVTQREAIQNHFLLCYSLLFCSDTSGLL
ORF3	–	3	177	64	114|37	MEESLPEPLGPGLAYYGNTERGYSKSFPPLLQPIILF

*ORF, open reading frame*.

**Table 2:** miRNAs that connected with hsa_circRNA_101833.

**ID**	**microRNA**	**MSA start**	**MSA end**	**Site type**
1	let-7a-2-3p/7g-3p	72	79	8mer-1a
2	let-7c-3p	73	79	7mer-1a
3	miR-101-3p.1	42	47	6mer
4	miR-128-3p/216-3p/3681-3p	43	49	7mer-1a
5	miR-136-5p	25	30	6mer
6	miR-144-3p	42	47	6mer
7	miR-144-5p	51	57	7mer-1a
8	miR-149-5p	80	86	7mer-1a
9	miR-27-3p	43	49	7mer-m8
10	miR-30-5p	38	44	7mer-m8
11	miR-3064-5p/6504-5p	80	86	7mer-1a
12	miR-3119	78	84	7mer-1a
13	miR-340-5p	66	71	6mer
14	miR-342-3p	45	50	6mer
15	miR-375	58	63	6mer
16	miR-3918	15	20	6mer
17	miR-4273/7156-5p	84	89	6mer
18	miR-4677-5p	84	89	6mer
19	miR-4685-5p/6837-5p	15	22	8mer-1a
20	miR-4797-5p	69	76	8mer-1a
21	miR-493-5p	73	79	7mer-1a
22	miR-5011-5p	91	96	6mer
23	miR-511-3p	87	93	7mer-1a
24	miR-513-3p	33	38	6mer
25	miR-513a-5p	44	49	6mer
26	miR-545-5p	40	46	7mer-m8
27	miR-5571-5p	63	69	7mer-1a
28	miR-5586-5p	18	23	6mer
29	miR-572	28	34	7mer-m8
30	miR-599	53	59	7mer-1a
31	miR-599	94	100	7mer-1a
32	miR-664-5p/4794	80	86	7mer-1a
33	miR-6834-3p	32	37	6mer
34	miR-6844	59	66	8mer-1a
35	miR-7113-5p	16	22	7mer-1a
36	miR-759	69	74	6mer

*MSA, Multiple sequence alignment*.

**Table 3:** Proteins that bind with hsa_circRNA_101833.

**ID**	**RBP**	**Details**
1	eIF4AIII_Human_GSE40778_HITS-CLIP	HHLE2_439259_eIF4AIII_rep2_439259_2
2	hnRNPC_Human_E-MTAB-1371_iCLIP	HIUHC_160199_HNRNPC_160199
3	hnRNPC_Human_E-MTAB-1371_iCLIP	HIUHC_160202_HNRNPC_160202
4	UPF1_Human_GSE47976_iCLIP	HIMP2_139591_UPF1_rep2_puromycoin_139591
5	eIF4AIII_Human_GSE40778_HITS-CLIP	HHLE2_439262_eIF4AIII_rep2_439262_17
6	AGO2_Human_GSE42701_HITS-CLIP	HHFKP_56722_cluster-8685_1_12
7	FUS_Human_GSE43308_HITS-CLIP	HHMF2_146682_FUS_rep2_1
8	UPF1_Human_GSE47976_iCLIP	HIMU2_172057_UPF1_rep2_untreated_172057
9	eIF4AIII_Human_GSE40778_HITS-CLIP	HHLE2_439261_eIF4AIII_rep2_439261_1
10	U2AF65_Human_E-MTAB-1371_iCLIP	HIUUC_189306_U2AF65_ctrl_189306
11	eIF4AIII_Human_GSE40778_HITS-CLIP	HHLE1_131383_eIF4AIII_rep1_131383_13
12	AGO2_Human_GSE42701_HITS-CLIP	HHFKP_56723_cluster-8685_2_12
13	eIF4AIII_Human_GSE40778_HITS-CLIP	HHLE1_131378_eIF4AIII_rep1_131378_1
14	U2AF65_Human_E-MTAB-1371_iCLIP	HIUUS_407000_U2AF65_ctrl_plus_HNRNPC_kd_407000
15	UPF1_Human_GSE47976_iCLIP	HIMP2_139590_UPF1_rep2_puromycoin_139590
16	PTB_Human_GSE42701_HITS-CLIP	HHFPT_109417_PTB_cluster-8685_7_9
17	AGO2_Human_GSE32109_PAR-CLIP	HPCB1_15634_G19662.1_68071959
18	hnRNPC_Human_E-MTAB-1371_iCLIP	HIUHC_160200_HNRNPC_160200
19	U2AF65_Human_E-MTAB-1371_iCLIP	HIUUS_406996_U2AF65_ctrl_plus_HNRNPC_kd_406996
20	hnRNPC_Human_E-MTAB-1371_iCLIP	HIUHC_160206_HNRNPC_160206
21	UPF1_Human_GSE47976_iCLIP	HIMU2_172058_UPF1_rep2_untreated_172058
22	U2AF65_Human_E-MTAB-1371_iCLIP	HIUUS_406993_U2AF65_ctrl_plus_HNRNPC_kd_406993
23	FUS_Human_GSE43308_HITS-CLIP	HHMF1_114226_FUS_rep1_6
24	hnRNPC_Human_E-MTAB-1371_iCLIP	HIUHC_160207_HNRNPC_160207
25	eIF4AIII_Human_GSE40778_HITS-CLIP	HHLE1_131379_eIF4AIII_rep1_131379_4
26	U2AF65_Human_E-MTAB-1371_iCLIP	HIUUS_406999_U2AF65_ctrl_plus_HNRNPC_kd_406999
27	U2AF65_Human_E-MTAB-1371_iCLIP	HIUUS_406998_U2AF65_ctrl_plus_HNRNPC_kd_406998
28	eIF4AIII_Human_GSE40778_HITS-CLIP	HHLE1_131380_eIF4AIII_rep1_131380_1
29	eIF4AIII_Human_GSE40778_HITS-CLIP	HHLE1_131382_eIF4AIII_rep1_131382_1
30	hnRNPC_Human_E-MTAB-1371_iCLIP	HIUHC_160204_HNRNPC_160204
31	hnRNPC_Human_E-MTAB-1371_iCLIP	HIUHC_160205_HNRNPC_160205
32	U2AF65_Human_E-MTAB-1371_iCLIP	HIUUS_406991_U2AF65_ctrl_plus_HNRNPC_kd_406991
33	PTB_Human_GSE42701_HITS-CLIP	HHFPT_109418_PTB_cluster-8685_8_10
34	U2AF65_Human_E-MTAB-1371_iCLIP	HIUUS_407001_U2AF65_ctrl_plus_HNRNPC_kd_407001
35	eIF4AIII_Human_GSE40778_HITS-CLIP	HHLE2_439260_eIF4AIII_rep2_439260_1
36	eIF4AIII_Human_GSE40778_HITS-CLIP	HHLE2_439258_eIF4AIII_rep2_439258_3
37	U2AF65_Human_E-MTAB-1371_iCLIP	HIUUC_189305_U2AF65_ctrl_189305
38	U2AF65_Human_E-MTAB-1371_iCLIP	HIUUS_406994_U2AF65_ctrl_plus_HNRNPC_kd_406994
39	UPF1_Human_GSE47976_iCLIP	HIMP2_139588_UPF1_rep2_puromycoin_139588
40	eIF4AIII_Human_GSE40778_HITS-CLIP	HHLE1_131381_eIF4AIII_rep1_131381_1
41	hnRNPC_Human_E-MTAB-1371_iCLIP	HIUHC_160201_HNRNPC_160201
42	U2AF65_Human_E-MTAB-1371_iCLIP	HIUUC_189304_U2AF65_ctrl_189304
43	eIF4AIII_Human_GSE40778_HITS-CLIP	HHLE2_439257_eIF4AIII_rep2_439257_18
44	UPF1_Human_GSE47976_iCLIP	HIMP2_139589_UPF1_rep2_puromycoin_139589
45	U2AF65_Human_E-MTAB-1371_iCLIP	HIUUC_189303_U2AF65_ctrl_189303
46	U2AF65_Human_E-MTAB-1371_iCLIP	HIUUS_406995_U2AF65_ctrl_plus_HNRNPC_kd_406995
47	U2AF65_Human_E-MTAB-1371_iCLIP	HIUUS_406992_U2AF65_ctrl_plus_HNRNPC_kd_406992
48	UPF1_Human_GSE47976_iCLIP	HIMP2_139592_UPF1_rep2_puromycoin_139592
49	ZC3H7B_Human_GSE38201_PAR-CLIP	HPLZC_25397_ZC3H7B_CID_009358_68059398
50	hnRNPC_Human_E-MTAB-1371_iCLIP	HIUHC_160203_HNRNPC_160203
51	UPF1_Human_GSE47976_iCLIP	HIMP2_139593_UPF1_rep2_puromycoin_139593
52	U2AF65_Human_E-MTAB-1371_iCLIP	HIUUS_406997_U2AF65_ctrl_plus_HNRNPC_kd_406997

*RBP, RNA binding protein*.

### GO and KEGG Pathway Analysis

**Figure 3:**
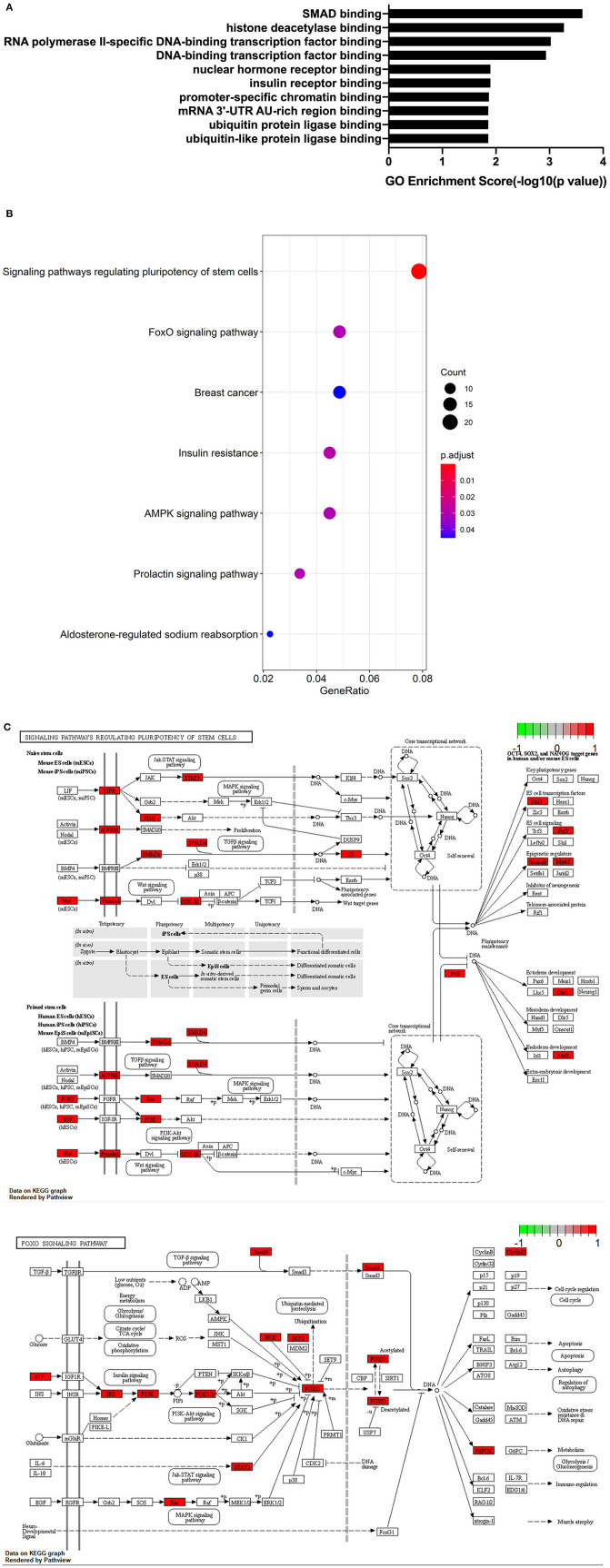
GO and KEGG analysis results. **(A)** GO annotations of target genes of microRNAs binding on hsa_circRNA_101833 with top 10 enrichment score encompassing the domains of physiological processes. **(B)** Dotplot of KEGG pathway analysis; the size of the circle represents the count of genes, and the color represents the *p*-values of pathways. **(C)** The visualization of signaling pathways regulating the pluripotency of stem cells and the FoxO signaling pathway conducted by Pathview.

After microRNAs binding on hsa_circRNA_101833 have been found, we searched the target genes of these microRNAs through TargetScan, miRDB, and miRTarBase (Supplementary Table 6). We finally enriched 19 GO terms, and all these terms were significantly different. The results revealed that the target genes of these microRNAs favored SMAD binding and histone deacetylase binding (Figure 3A). Among them, SMAD binding may be more likely to progress in which hsa_circRNA_101833 participates in the formation of IAs. According to several studies, the SMAD family is related to the formation of thoracic aortic aneurysm (Regalado et al., 2011; Mao et al., 2012; Wang et al., 2016), and animal experiments have proven that the deficiency of SMAD3 would promote the formation of thoracic aortic aneurysm (Dai et al., 2015). Also, KEGG pathway analysis has been performed. Depending on our annotation, the target genes of these microRNAs are associated with signaling pathways regulating the pluripotency of stem cells, insulin resistance, FoxO signaling pathway, Prolactin signaling pathway, AMPK signaling pathway, breast cancer, and aldosterone-regulated sodium reabsorption (Figure 3B). Among them, the signaling pathways regulating the pluripotency of stem cells and the FoxO signaling pathway contain the TGF-β signaling pathway and the MAPK signaling pathway (Figure 3C), and both of them are well-studied pathways related to the pathological processes of IAs (Weinsheimer et al., 2007; Yamashita et al., 2008).

## Discussion

In our study, we revealed hundreds of differentially expressed circRNAs between human IA tissues and STA tissues. Based on their expressive intensity and other criteria as mentioned before, five upregulated circRNAs were selected; finally, only hsa_circRNA_101833 overexpressed significantly after being verified by qRT-PCR. With GO analysis of the target genes of microRNAs binding on hsa_circRNA_101833, hsa_circRNA_101833 might participate in the pathological processes of IAs, especially through the way of SMAD binding.

The SMAD family contains three subfamilies: the five receptor-activated SMADs (R-SMADs), the one common mediator SMAD (Co-SMAD), and the two inhibitory SMADs (I-SMADs) (Moustakas et al., 2001; Derynck and Zhang, 2003; Shi and Massague, 2003). Smad2 and 3 are signals for TGF-β (transforming growth factor-β) (Flanders, 2004). TGF-β has a lot of functions, acts on a variety of different cells, and regulates many distinctive complex intracellular functions. And several studies suggest that TGF-β plays a critical role in the pathological processes of IAs (Yamashita et al., 2008; Carta et al., 2009), and the interactions between R-SMAD (SMAD2 and SMAD3) and Co-SMAD (SMAD4) regulate the canonical pathway that TGF-β attends (Akhurst, 2012). Numerous studies have proven the relationship between SMAD and aneurysm. Regalado et al. (2011) showed that SMAD3 mutations are responsible for 2% of familial thoracic aortic aneurysms and dissections, and aneurysms resulting from the SMAD3 mutation involve different arteries, including intracranial arteries. What is more, a study about the association between SMAD3 gene and IA (Liao et al., 2018) demonstrates that SMAD3 gene polymorphisms were significantly related to IAs. Apart from SMAD3, the relationship between SMAD4 and aneurysms also has been studied. Mao et al. (2012) found that silencing SMAD4 of the vascular smooth muscle cell (VSMC) would result in vascular defects by decreasing VSMC differentiation, proliferation, migration, as well as cell attachment and spreading. And the differentiation and migration of VSMC may be one of the mechanisms that contribute to the formation of aneurysm. Furthermore, we performed a KEGG pathway analysis to annotate the target genes of microRNAs binding on hsa_circRNA_101833. Our results showed that these genes associated with the signaling pathways regulating the pluripotency of stem cells and the FoxO signaling pathway that are involved in the formation of IA. Both GO enrichment and KEGG analysis suggested that upregulation of hsa_circRNA_101833 may promote the formation of IA.

We detected hundreds of differentially expressed circRNAs using the Arraystar human circRNA Microarray; 456 (83.82%) of the upregulated circRNAs and 349 (87.69%) of the downregulated circRNAs belong to the exonic type. This constitution of circRNAs is in common with previous studies (Cordes et al., 2009; Merk et al., 2012; Zhang et al., 2013). We also found that circRNAs can adsorb microRNAs and interact with different proteins. These findings are all in accordance with previous studies (Hansen et al., 2013; Du et al., 2017). Besides, the function of hsa_circRNA_101833 has been analyzed in human IA tissues for the first time. We revealed that the ORF of hsa_circRNA_101833 may encode the TIM superfamily, but the relationship between TIM and aneurysm is still unknown, so we cannot determine whether hsa_circRNA_101833 influences the pathological process through its expression product. Inevitably, there are several limitations in this study. First of all, our tissue sample size is small. Our results need a larger number of IA samples to testify. Secondly, because aneurysm samples are rare, some of our samples were stored in liquid nitrogen for several weeks; perhaps this would affect the amount of circRNAs. Thirdly, this study is mainly a bioinformatic analysis. Further studies should be done to prove the function of hsa_circRNA_101833 in the aneurysm pathological process. Lastly, due to the lack of studies about circRNAs in aneurysm, we cannot compare our results with others in order to enhance our methods.

In general, hundreds of differentially expressed circRNAs using the Arraystar human circRNA Microarray were detected in IAs as compared to the STA tissue. The up-regulation of hsa_circRNA_101833 was associated with the formation of IAs through the impact on the SMAD family or participating the TGF-β sigaling pathway and MAPK signaling pathway

## Data Availability Statement

The datasets presented in this study can be found in online repositories. The names of the repository/repositories and accession number(s) can be found in the article/Supplementary Material.

## Ethics Statement

The studies involving human participants were reviewed and approved by Institutional review board of the Beijing Tiantan hospital. The patients/participants provided their written informed consent to participate in this study.

## Author Contributions

SW and HW were in charge of supervising the whole study. XC contributed to the conception or design of the work. XC and SY were responsible for drafting and revising. QL, ML, JW, and JY were responsible for analysis and interpretation of data. All authors contributed to manuscript revision, read, and approved the submission.

## Conflict of Interest

The authors declare that the research was conducted in the absence of any commercial or financial relationships that could be construed as a potential conflict of interest.
