# Supplementary material for: Circular RNA circDUS2 Is a Potential Biomarker for Intracranial Aneurysm
Source: Front Aging Neurosci. 2021 May 19;13:632448. doi: 10.3389/fnagi.2021.632448 (PMC8171118; doi:10.3389/fnagi.2021.632448)
Supplement: Supplementary file 1 [file Table_1.docx]

Supplementary Material

Supplementary Table 1:RNA quantification and quality assurance by NanoDrop ND-1000

| **Sample ID OD 260/280 OD 260/230 Conc Volume Quantity QC result**  **Ratio Ratio (ng/μL) (μL) (ng) Pass or Fail** |
| --- |
| STA1 1.84 2.33 366.28 15 5494.20 Pass  STA2 1.84 2.39 178.16 15 2672.40 Pass  STA3 1.90 2.08 408.23 15 6123.45 Pass  STA4 1.86 2.23 405.50 15 6082.50 Pass  STA5 1.92 2.17 720.48 15 10807.20 Pass  AN1 1.83 2.38 311.20 15 4668.00 Pass  AN2 1.91 2.18 625.76 15 9386.40 Pass  AN3 1.90 2.34 619.68 60 37180.80 Pass  AN4 1.88 2.21 231.37 15 3470.55 Pass  AN5 1.92 2.30 560.15 30 16804.50 Pass |

*For spectrophotometer, the O.D. A260 /A280 ratio should be close to 2.0 for pure RNA (ratios between 1.8 and 2.1 are acceptable). The O.D. A260/A230 ratio should be more than 1.8.

Supplementary Table 2:Labeling Efficiency

| **Sample ID Dye Dye cRNA concentration Specific activity* Volume Total amount** **name pmol/μL (μg/μL) (pmol dye/μg cRNA) (μL) (μg)** |
| --- |
| STA1 Cy3 11.32 0.41579 27.22528 10 4.1579  STA2 Cy3 10.25 0.36381 28.17405 10 3.6381  STA3 Cy3 12.83 0.45823 27.99904 10 4.5823  STA4 Cy3 10.31 0.42861 24.05450 10 4.2861  STA5 Cy3 12.60 0.42668 29.53033 10 4.2668  AN1 Cy3 11.92 0.61466 19.39284 10 6.1466  AN2 Cy3 12.01 0.63992 18.76797 10 6.3992  AN3 Cy3 13.08 0.64829 20.17616 10 6.4829  AN4 Cy3 11.14 0.5862 19.00375 10 5.862  AN5 Cy3 13.13 0.7341 17.88585 10 7.341 |

*For two-color, if the yield is <825 ng and the specific activity is <8.0 pmol Cy3 or Cy5 per μg cRNA do not proceed to the hybridization step. Repeat cRNA preparation.

*For one-color, if the yield is <1.65 μg and the specific activity is <9.0 pmol Cy3 or Cy5 per μg cRNA do not proceed to the hybridization step. Repeat cRNA preparation.

Supplementary Table 3: qRT-PCR primer sequences

| Primer name | Primer F (5'-3') | Primer R(5'-3') |
| --- | --- | --- |
| hsa_circRNA_104172 | CTCTCCCATCATCATCTTGAATT | GCTCCCAACTAGAAAGTATCTCTT |
| hsa_circRNA_048764 | GCTGCTGCCAAGAAAGACT | GCGGGAAACTTCTGTCAAG |
| hsa_circRNA_037798 | TTGGTGACAGAGCGAGAGCTA | TGGGATGGAGCACATTTTATAG |
| hsa_circRNA_406748 | CAGTGGAGAGAGTTTGTCCATCT | CTCAAAGGTGACTGGCTTCTGA |
| hsa_circRNA_101833 | CGGACATTGTTTACTGTGAGAT | ATGTGTTCTGGCTTTGTACAGA |

Supplementary Table 4: circRNAs expression profiling data.

Supplementary Table 5: Significant differentially expressed circRNAs (FC≥1.5 and P≤0.05)

Supplementary Table 6: Target genes of microRNAs binding on hsa_circRNA_101833
